# Deep causal learning for robotic intelligence

**DOI:** 10.3389/fnbot.2023.1128591

**Published:** 2023-02-22

**Authors:** Yangming Li

**Affiliations:** RoCAL, Rochester Institute of Technology, Rochester, NY, United States

**Keywords:** deep causal learning, robotic perception, complementary perception, robotics, intelligence

## Abstract

This invited Review discusses causal learning in the context of robotic intelligence. The Review introduces the psychological findings on causal learning in human cognition, as well as the traditional statistical solutions for causal discovery and causal inference. Additionally, we examine recent deep causal learning algorithms, with a focus on their architectures and the benefits of using deep nets, and discuss the gap between deep causal learning and the needs of robotic intelligence.

## 1. Introduction

Intelligent robots infer knowledge about the world from sensor perception, estimate status, model the world, and plan and execute tasks. Although intelligent robots have achieved remarkable progress in the past two decades, improving their reliability in the real world remains a challenge. The challenges are rooted in the wide variance of environments and robotic tasks and the uncertainties of the world, sensor observation, models, status, and the execution of tasks.

Robots achieve intelligence through the use of knowledge or methods that learn knowledge from data. Early intelligent robots and some state-of-art applied intelligent robots are programmed with knowledge and rules for making decisions based on dynamic observations. This type of achieved intelligence has predictable performance and is explainable; however, it often lacks adaptiveness as the program complexity grows exponentially with respect to the complexity of tasks. Additionally, robots can use artificial intelligence (AI) algorithms (to learn from data) to achieve intelligence. These methods are considered similar to how humans achieve intelligence and are considered a possible solution that breaks the bottleneck of applicability, scalability, and online learning. However, as today's AI methods demonstrate superior performance in terms of “interpolating” data, these methods still have difficulty in finding knowledge and reasoning.

Humans, or even animals, effortlessly understand the world from an early age and build up prior knowledge quickly to make causal decisions in daily life. We can use the perception of an object's physical properties as an example. Even infants demonstrate instinctual behavior when inspecting a new toy with their hands and eyes in tandem with learning the toy's properties (Smith et al., 2020). Robots, in comparison, still have problems understanding and operating the most commonly used objects in daily life. Although it is clear that causality is critical from low-level visual perception to high-level decision-making, state of art robots rarely establish the causal relationship and utilize it to improve intelligence. However, there are examples of “causal” relationships improving robotic intelligence. For example, simultaneous localization and mapping (SLAM) explicitly utilizes the fact that causal relationships of robot movements causally change observations and use a Bayesian network to improve mapping and localization simultaneously (Thrun et al., 2005).

Causal learning consists of causal discovery and causal inference. Classical causal learning methods include causal discovery, which learns cause-effect relationships, and causal inference, which estimates the level of impact that changes in factors have on each other. Traditional causal learning algorithms mainly use statistical theories and tools. With the development of deep-learning technology, there are trends that use deep learning to improve causal learning with high-dimensional data and big data and trends that use causal learning to improve deep-learning model expandability, extrapolation capability, and explainability. Although these emerging techniques are not developed and have not yet been tested on intelligent robots, they do have great potential to improve robotic intelligence and expand the applicability of intelligent robots. This paper incompletely but systematically reviews causal cognition, causal learning, and deep causal learning, and discusses the need for deep causal learning in robotic intelligence. The rest of the Review is organized as follows: the section “Causal Cognition and Intelligence” briefly introduces causal cognition from the psychological perspective, the section “Causal Learning” presents statistical causal discovery and causal inference, the “Deep Causal Learning” section discusses deep causal learning for robotic intelligence, and the final section presents conclusions.

## 2. Causal cognition and intelligence

Although there is much debate about the mechanism of cognition, modern physiological studies generally support that the way in which humans cognize causal regularities is more sophisticated than that of any other animal on the planet (Penn and Povinelli, 2007). Causal cognition has major differences with associative learning, as it can improve inferences from non-obvious and hidden causal relationships (Kuhn, 2012). Learning causal relationships is critical for humans (Spelke et al., 1992; Kuhn, 2012), as it confers an important advantage for survival (Legare et al., 2017; Bender and Beller, 2019).

It is clear that to achieve or avoid an outcome, one may want to predict the probability that an effect will occur given that a certain cause of the effect occurs. It remains unclear as to how the input non-causal empirical observations of cues and outcomes yield output values. Studies have shown that causal cognition emerges early in development (Bender and Beller, 2019). Researchers are amazed by how children learn so much about the world so quickly and effortlessly (Gopnik et al., 2004; Sobel and Legare, 2014). Studies have demonstrated that infants as young as 4.5 months register particular aspects of physical causality (Leslie and Keeble, 1987; Spelke et al., 1992; Needham and Baillargeon, 1993; Hespos and Baillargeon, 2001), toddlers recognize various causal relations in the psychological domain, especially about others' desires and intentions (Wellman, 1992; Bonawitz et al., 2010), and preschoolers understand that biological and psychological events can rely on non-obvious hidden causal relations (Gelman and Wellman, 1991; Tooby et al., 1994). Adults use substantive prior knowledge about everyday physics and psychology to make new causal judgments (Ahn et al., 2000).

## 3. Causal learning

As presented in the previous section, causal learning is associated with human intelligence and has been widely studied. Traditional causal learning uses statistical methods to discover knowledge from data and perform causal inference. These methods are widely used in the field of medical science, economics, epidemiology, etc, but are rarely used in the domain of intelligent robotics (Guo et al., 2020; Yao et al., 2021a).

### 3.1. Causal discovery

Causal discovery learns the causal structure that represents the causality between observations X, treatments t, and outcomes y.

Traditional causal discovery relies on statistically verifying potential causal relationships or estimating functional equations to establish causal structures. Generally, there are four types of representative algorithms for traditional causal discovery: constraint-based algorithms and the score-based algorithms, which rely on statistical verification, functional causal model-based algorithms, which rely on functional estimation, and hybrid algorithms, which fuse multiple algorithms (Guo et al., 2020).

#### 3.1.1. Constraint-based algorithms

Constraint-based algorithms analyze conditional independence in observation data to identify causal relationships. This family of algorithms often uses statistical testing algorithms to determine the conditional independence of two variables, given their neighbor nodes, then further determine the direction of the causality.

Mathematically, we can use three variables *X*, *Y*, and *Z* to explain constraint-based algorithms. The causal relationship is verified by conditional independence, for example, *X* ⫫ *Y* | *Z*, which is equivalent to zero conditional information *I*[*X*; *Y* | *Z*] = 0. This is defined as faithfulness in causal learning, as explained in Definition 3.1. If the three variables are discrete, *χ*^2^ and *G*^2^ can verify the conditional independence based on the contingency table of *X*, *Y*, and *Z*. If the three variables are linear and multivariate Gaussian, we can verify the conditional indecency by a test if the partial correlation is zero. For other circumstances, it often needs extra assumptions to ensure the verification is computationally tractable.

Definition 3.1. *(Faithfulness). Conditional independence between a pair of variables*, $x_{i}⫫x_{j}|x^{-}$ *for* $x_{i}\neq x_{j},x^{-}\subseteq X\backslash \left\{x_{i},x_{j}\right\}$*, can be estimated from a dataset X iff* *x*^−^ *d-separates* *x*_*i*_ *and* *x*_*j*_ *in the causal graph* G=(V,E).

The conditional independence is symmetric, and additional tests are required to determine the orientations of edges. When *X* ⫫ *Y* | *Z*, there are three possible graphical structures, including two chains (*X* ← *Z* ← *Y* and *X* → *Z* → *Y*) and a fork *X* ← *Z* → *Y*. The determination of which structures are induced based on the adjacency among variables, the background knowledge, etc. When *X* ⫫ *Y* | *Z* it is a collision structure (*X* → *Z* ← *Y*).

Constraint-based algorithms use assumptions to improve efficiency and effectiveness for causal discovery from data. For example, the Peter-Clark algorithm assumes i.i.d. sampling and no latent confounders, which prunes edges between variables by testing conditional independence based on observation data, and determines and propagates the orients to form the directed acyclic graph (DAG) (Spirtes et al., 2000). The inductive causation algorithm assumes stable distributions (Definition 3.2), tests conditional independence to find the associative relationship between variables, finds collision structures, determines orients based on a variable's adjacency, and propagates directions (Pearl, 2009).

Definition 3.2. *(Stable distribution). A distribution is stable if a linear combination of two independent random variables with this distribution has the same distribution, up to location and scale parameters*.

Other constraint-based algorithms focus on weakening assumptions, thereby extending causal discovery methods to other distribution families (Sejdinovic et al., 2013; Ramsey, 2014; Glymour et al., 2019), causal discovery from data with unobserved confounders (Spirtes et al., 2013; Guo et al., 2020).

#### 3.1.2. Score-based algorithms

Score-based algorithms learn causal graphs by maximizing the goodness-of-fit test scores of the causal graph *G* given observation data *X* (Spirtes et al., 2000). Because these algorithms replace the conditional independence tests with the goodness-of-fit tests, they relax the assumption of faithfulness (Definition 3.1) but often increase computational complexity. This is because the scoring criterion *S*(*X, G*) enumerates and scores the possible graphs under parameter adjustments. For example, the popular Bayesian information criterion adopts the score function $S\left(X,G\right)=\log P\left(X|\hat{\theta },G\right)-J/2\log\left(n\right)$ to find the graph that maximizes the likelihood of observing the data, while the number of parameters and the sample size is regularized, where $\hat{\theta }$ is the maximum likelihood estimation of the parameters and *J* and *n* denote the number of variables and the number of instances, respectively (Schwarz, 1978).

It is not tractable to score all possible causal graphs given observation data because it is NP-hard (Chickering, 1996) and NP-complete (Chickering, 1996). In practice, score-based algorithms use heuristics to find a local optimum (Chickering, 2002; Ramsey et al., 2017). For example, the greedy equivalence search algorithm uses Bayesian-Dirichlet equivalence score *S*_*BD*_:


(1) $$\begin{array}{l} S_{DB}(X,G)=\log\prod_{j\;=\;1}^{J}0.001^{(r_{j}-1)q_{j}}\prod_{k\;=\;1}^{q_{j}}\frac{\text{Γ}(10/q_{j})}{\text{Γ}(10/q_{j}+N_{jk})}\;\\ \;\prod_{l\;=\;1}^{r_{j}}\frac{\text{Γ}(10/r_{i}/q_{i}+N_{jkl})}{\text{Γ}(10/r_{i}/q_{i})}, \end{array}$$

To score a graph *G*, where *r*_*j*_ and *q*_*j*_ signify the numbers of configurations of variable *x*_*j*_ and the numbers of configurations of parent set *Pa*(*x*_*j*_), respectively, Γ(·) denotes the gamma function, *N*_*jkl*_ denotes the number of records of *x*_*j*_ = *k*, and *Pa*(*x*_*j*_) is in the *k*-th configuration.

Widely used score-based algorithms optimize the searching and scoring process based on assumptions, such as linear-Gaussian models (Fukumizu et al., 2007), discrete data (Heckerman et al., 1995), and sparsity (Zheng et al., 2020). Additionally, there are studies on relaxing the assumptions for causal discovery from non-linear and arbitrarily distributed data (Huang et al., 2018). Compared with constraint-based algorithms, score-based algorithms can compare the output models in the space searched for model selection.

#### 3.1.3. Functional causal models-based algorithms

Functional causal models-based algorithms represent the causal relationship with functional equations (Define 0.0.3).

Definition 3.3 *(Functional equation). A functional equation represents a direct causal relation as* *y* = *f*_θ_(*X, n*)*, where* *X* *is the variables that directly impact the outcome* *y*, *n* *is noise with* *n* ⫫ *X, and* *f*_θ_ *is the general form of a function*.

Causal discovery with functional equations can be expressed as sorting causal orders (which variables depend on which) from observation data. We use a linear non-Gaussian acyclic model to explain the process with a simple linear case **x** = *A***x** + μ, where **x** denotes the variable vector, A denotes the adjacency matrix, and μ denotes the noise independent of **x**. With this representation, the causal discovery is the equivalent of estimating a strictly lower triangle matrix *A* that determines the unique causal order *k*(*x*_*i*_), ∀*x*_*i*_ ∈ *mathbfx*, which can be performed in the form of matrix permutation as described by Shimizu et al. (2006).

Functional causal models-based algorithms have demonstrated effectiveness in producing unique causal graphs. For example, the post-non-linear causal model learns the causal relationship that can be represented by a post-non-linear transformation on a non-linear effect of the cause variables and additive noises (Zhang and Hyvarinen, 2012). This algorithm can be further improved with independent component analysis (Taleb and Jutten, 1999) and relaxed by a warped Gaussian process, with the noise modeled by the mixture of Gaussian distributions (Zhang et al., 2015). Compared with the constraint-based and score-based algorithms, functional causal models-based algorithms are able to distinguish between different DAGs from the same equivalent class.

#### 3.1.4. Hybrid algorithms

Hybrid algorithms combine multiple algorithms to overcome problems that exist in constraint-based or score-based algorithms. For example, Tsamardinos et al. (2006) use the max-min parents and children algorithm (constrained-based) to learn the skeleton of the causal graph and uses a Bayesian scoring hill-climbing search (score-based) to determine the orients of edges. Wong et al. (2002) use the conditional independence test to learn the skeleton of the causal graph and use a metric to search good network structures.

### 3.2. Causal inference

Causal inference is the process of estimating the changes of outcomes **y** given treatments **t**. Before we discuss causal inference algorithms, we need to first define the metrics (Definition 3.4) for measuring causal inference. ATE, ATT, CATE, and ITE measure the treatment effects of the population, the treated group, a subgroup of a given feature *x*, and individuals, respectively.

Definition 3.4. *(Treatment Effect)*.

- *Average Treatment Effect (ATE):* ATE = *E*[*Y*(*w* = 1) − *Y*(*w* = 0)].
- *Average Treatment Effect on the Treated Group (ATT):* ATT = *E*[*Y*(*w* = 1) | *w* = 1] − *E*[*Y*(*w* = 0) | *w* = 1].
- *Conditional Average Treatment Effect (CATE):* CATE = *E*[*Y*(*w* = 1) | *X* = *x*] − *E*[*Y*(*w* = 0)|*X* = *x*].
- *Individual Treatment Effect (ITE):* ITE_*i*_ = *Y*_*i*_(*w* = 1) − *Y*_*i*_(*w=0*).

Causal inference estimates the treatment effects for specific groups. However, the different distributions of groups and the existence of confounders make the task very challenging. According to the methodological differences, existing classical algorithms for addressing these problems can be grouped into reweighting-based algorithms, stratification-based algorithms, batching-based algorithms, and tree-based algorithms.

#### 3.2.1. Reweighting-based algorithms

Reweighting-based algorithms assign appropriate weights to the samples to create pseudo-populations or reweight the covariates to mitigate the differences in the distributions between the treated groups and the control groups. These algorithms are designed to address the selection bias between the treated groups and the control groups.

Both sample and covariate reweighting are used to address selection bias. The inverse propensity weighting algorithm is one of the pioneering works on reweighting samples. This algorithm uses propensity scores (Definition 3.5) to find the appropriate weights for samples as *r* = *T*/*e*(*x*) + (1 − *T*)/(1 − *e*(*x*)), where *T* is the treatment.

Definition 3.5. *(Propensity Score). Propensity score* *e*(*x*) *is the conditional probability of assignment to a particular treatment given a vector of observed covariates* *e*(*x*) = *Pr*(*T* = 1|*X* = *x*).

**Figure 1:**
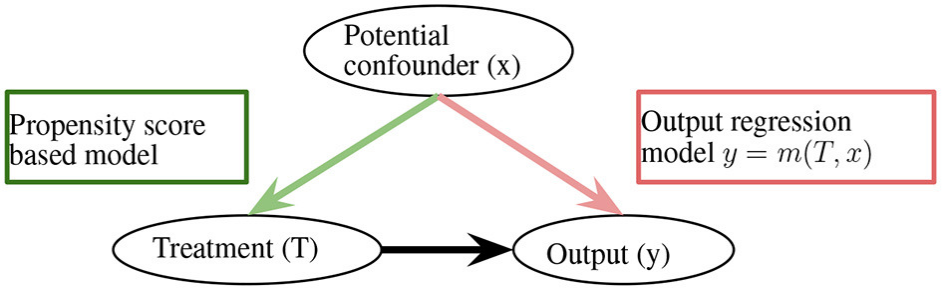
Doubly robust estimator. *m*(·) can be an arbitrary function.

With the reweighting, the ATE is defined as $A\hat{T}E=1/N_{T}\sum_{i=1}^{N_{T}}T_{i}Y_{i}^{F}/\hat{e}(x)-1/N_{C}\sum_{i=1}^{N_{C}}\left(1-T_{i}\right)Y_{j}^{F}/(1-\hat{e}(x))$. This method is sufficient for removing bias; however, it heavily relies on the correctness of propensity scores (Robins et al., 1994). Along the lines of propensity score-based sample reweighting, the doubly robust estimator combines the propensity score weighting with the outcome regression to remain unbiased, as long as the propensity score or outcome regression is correct (Figure 1; Robins et al., 1994), the overlap weights algorithm solves the extreme propensity score problem by reducing the weights of the unites that locate in the tails of the propensity score distributions (Li F. et al., 2019).

The covariate reweight algorithms learn sample weights from data through regression. To reweight a covariate, Hainmueller (2012) uses a maximum entropy reweighting scheme to calibrate sample weights to match the moments of the treated group and the control group and minimizes information loss by keeping weights close to the base weights.

Additionally, there are algorithms that balance distributions with both covariate and sample reweighting. The covariate balancing propensity score estimates the propensity score by solving $E\left[W_{i}{\tilde{x}}_{i}/e\left(x_{i};\beta \right)+\left(1-W_{i}\right){\tilde{x}}_{i}/\left(1-e\left(x_{i};\beta \right)\right)\right]$ to measure the probability of being treated and the covariate balancing score and improves the empirical performance of propensity score matching (Imai and Ratkovic, 2014). Data-driven variable decomposition (D2VD) balances distribution by automatically decomposing observed variables confounders, adjusted variables, and irrelevant variables (Kuang et al., 2017b). Differentiated confounder balancing (DCB) selects and differentiates confounders and reweights both the sample and the confounders to balance distributions (Kuang et al., 2017a).

#### 3.2.2. Stratification-based algorithms

Stratification-based algorithms split observations into subgroups, which are similar under certain measurements. With subgroups that have balanced distributions, ATE is estimated as ${\hat{\tau }}^{\text{strat}}=\sum j=1Jq\left(j\right){Ȳ}_{t}\left(j\right)-{Ȳ}_{c}\left(j\right)$. For example, if a model can predict the strata in which subjects always stay in the study regardless of which treatment they were assigned, then the data from this strata is free of selection biases (Frangakis and Rubin, 2002; Jin and Rubin, 2008). The stratification can be performed on samples on the basis of the propensity score to improve the estimation robustness, as explained in the marginal mean weighting through stratification algorithm (Hong, 2010). Additionally, the stratification algorithms can be combined with propensity score-based algorithms to preprocess data to remove imbalances of pre-intervention characteristics (Linden, 2014).

#### 3.2.3. Matching-based algorithms

Matching-based algorithms use specific distance measurements to match samples in the treatment group with ones in the control group to estimate the counterfactuals and reduce the estimation bias of confounders. Matching-based algorithms require the definition of distance metrics and the selection of matching algorithms. Euclidean distances and Mahalanobis distances are commonly used as distance metrics in the original data space, while transformations, such as propensity score-based transformation, and observed outcome information are commonly used in the transformed feature space (Stuart, 2010; Yao et al., 2021a). For matching algorithms, nearest neighbor, Caliper, stratification, and kernel-based methods are all widely adopted (Guo et al., 2020). It is worth noticing that matching-based algorithms can be used in data selection, as well as experimental design and performance. The latter uses matching to identify subjects that have outcomes that should be collected (Kupper et al., 1981; Reinisch et al., 1995), which potentially reduces costs and the difficulty in collecting effective data.

#### 3.2.4. Tree-based methods

A tree structure naturally divides data into disjoint subgroups. Although the subgroups have similar *e*(*x*), the estimation of the treatment effect is unbiased. Bayesian additive regression trees (BART), a Bayesian “sum-of-trees” model, is a flexible approach for fitting a variety of regression models while avoiding strong parametric assumptions. With BART, the treatment *y* is the sum of subgroups as *y* = *g*(*x*; *T*_1_, θ_1_) + ⋯ + *g*(*x*; *T*_*n*_, θ_*n*_)+σ, where σ is Gaussian white noise (Chipman et al., 2010). Similarly, the classification and regression trees (CART) algorithm also splits data into classes that belong to the response variable. CART is different from BART in that it recursively partitions the data space and fits a simple prediction model for each partition (Loh, 2011). Causal forests ensemble multiple causal trees to achieve a smooth estimation of CATE. Causal forests are based on Breiman's random forest algorithm and maximize the difference across splits in the relationship between an outcome variable and a treatment variable to reveal how treatment effects vary across samples (Wager and Athey, 2018).

## 4. Deep causal learning

Deep learning (DL) has successfully attracted researchers from all fields as it demonstrates the power and the simplicity of learning from data (Goodfellow et al., 2016). The majority of existing DL algorithms use specialized architecture to establish end-to-end relationships from observation data, e.g., convolutional neural networks (CNN) for data with spatial locality, recurrent neural networks (RNN) for data with sequential or temporal structure, transformers for data with context information, autoencoders for data that need compressed representation, and generative adversarial networks for data that need domain adaption (LeCun and Bengio, 1995; Graves, 2012; Kingma and Welling, 2013; Vaswani et al., 2017; Goodfellow et al., 2020). Despite the remarkable success DL has achieved, some challenges remain, such as model expandability, extrapolation capability, and explainability. Causal learning (CL), on the other hand, discovers knowledge, explains prediction, and has extendable structures, but struggles with high dimensional data and scalability problems. Therefore, complementing DL with CL, and vice versa, can be a way forward. Actually, recent studies have made great progress and have demonstrated the advantages of deep causal learning in that prior knowledge can be used to disentangle modeling problems and reduce data needs (Parascandolo et al., 2018; Bengio et al., 2019; Yang et al., 2020), it has superior performance at extrapolating unseen data (Mart́ınez and Marca, 2019; Pawlowski et al., 2020a), can modularize learning problems, incrementally learns from multiple studies (Kaushik et al., 2019; Singla et al., 2019; Pawlowski et al., 2020b), and demonstrates potential as a solution to artificial general intelligence (Pearl, 2009; Guo et al., 2020).

Below, we introduce some of the representatives in plain language, with a focus on the network architecture and the benefits of using the architecture. Because the algorithms we review share many common characteristics, such as most of them using two or more neural networks and most of the representation learning involving CNN, we categorize the algorithms into the following categories to maximize the uniqueness among the categories.

### 4.1. Using DL for learning representation

Balancing neural networks and balancing linear regression are pioneering works that use deep neural networks to solve the problem of causal learning from high dimensional data (Johansson et al., 2016). These algorithms learn a representation *g* : *X* → ℝ^*d*^ through deep neural networks or feature reweighting and selection, then based on the features *g*(*X*), learn the causal effect *h* : ℝ^*d*^ × *T* → ℝ. These models learn balanced representations that have similar distributions among the treated and untreated groups and demonstrate effectiveness in cases that have one treatment.

Similarity-preserved individual treatment effect (SITE) uses two networks to preserve local similarity and balances data distributions simultaneously (Yao et al., 2018). The first network is a representation network, which maps the original pretreatment covariate space **X** into a latent space **Z**. The second network is a prediction network, which predicts the outcomes based on the latent variable **Z**. The algorithm uses position-dependent deep metric and middle point distance minimization to enforce two special properties on the latent space Z, including the balanced distribution and preserved similarity. The adaptively similarity-preserved representation learning method for causal effect estimation (ACE) preserves similarity in representation learning in an adaptive way to extract fine-grained similarity information from the original feature space and minimizes the distance between different treatment groups, as well as the similarity loss during the representation learning procedure (Yao et al., 2019a). ACE applies balancing and adaptive similarity preserving (BAS) regularization to the representation space. The BAS regularization consists of distribution distance minimization and adaptive pairwise similarity preservation, thus decreasing the ITE estimation error.

Johansson et al. (2018) presented a theory and an algorithmic framework for learning to predict outcomes of interventions under shifts in design changes in both intervention policy and feature domain. This framework combines representation learning and sample reweighting to balance source and target designs, emphasizing information from the source sample relevant to the target. As a result, this framework relaxes the strong assumption of having a well-specified model or knowing the policy that gave rise to the observed data.

### 4.2. End-to-end deep causal inference

Shalit et al. (2017) proposed treatment-agnostic representation networks (TARNETs) to estimate ITE based on the Rubin potential outcomes framework under the assumption of strong ignorability. This algorithm uses integral probability metrics to measure distances between distributions and derives explicit bounds for the Wasserstein and maximum mean discrepancy (MMD) distances. Therefore, this algorithm is an end-to-end regularized minimization procedure that fits the balanced representation of the data and a hypothesis for the outcome. Based on this work, Hassanpour and Greiner (2019a) proposed a context-aware importance sampling reweighting scheme to estimate ITEs, which addresses the distributional shift between the source (outcome of the administered treatment appearing in the observed training data) and target (outcome of the alternative treatment) that exists due to selection bias. Perfect matching augments samples within a minibatch with their propensity-matched nearest neighbors to improve inference performance in settings with many treatments (Schwab et al., 2018). Perfect matching is compatible with other architectures, such as the TARNET architecture, and extends these architectures to any number of available treatments. Additionally, perfect matching uses the nearest neighbor approximation of precision in the estimation of heterogenous effects with multiple treatments to select models without requiring access to counterfactual outcomes.

Alaa et al. (2017) modeled the inference of individualized causal effects of a treatment as a multitask learning problem. The algorithm uses a propensity network and a potential outcomes network to estimate ITE (Definition 3.4). The propensity network is a standard feedforward network and is trained separately to estimate the propensity score *e*(*x*_*i*_) (Definition 3.5) from (*x*_*i*_, *t*_*i*_). By assigning “simple models” to subjects with very high or very low propensity scores (*e*(*x*_*i*_) close to 0 or 1), and “complex models” to subjects with balanced propensity scores (*e*(*x*_*i*_) close to 0.5), it alleviates the selection bias problem. The potential outcomes network is a multitask network that models the potential outcomes $E[Y_{i}^{(1)|x_{i}}$ and $E[Y_{i}^{(0)|x_{i}}$ as two separate but related learning tasks; therefore, the treatment assignments and the subjects' characteristics are fully utilized.

### 4.3. Autoencoder-based algorithms

**Figure 2:**
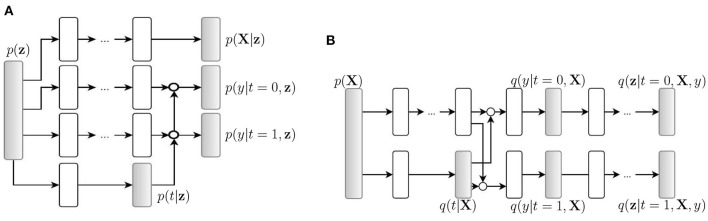
Causal effect variational autoencoder (Louizos et al., 2017). *q*(·) is a tractable distribution that approximates the true distribution *p*(·). **X**, **t**, and **y** are observations, treatments, and outcomes, respectively. **(A)** Model net. **(B)** Inference net.

Causal effect variational autoencoder (CEVAE) uses variational autoencoder (VAE) structures to estimate individual treatment effects (Louizos et al., 2017). The algorithm uses an inference network (Figure 2B) and a model network (Figure 2A) to simultaneously estimate the unknown latent space summarizing the confounders and the causal effect, based on latent variable modeling. Because the algorithm uses the two networks to utilize both the causal inference with proxy variables and latent variable modeling, its performance is competitive with the state-of-the-art methods on benchmark datasets and has improved robustness on the problems with hidden confounders.

The deep-treat algorithm uses two networks for constructive effective treatment policies by addressing the problems of the biased observed data and unavailable counterfactual information (Atan et al., 2018). The first network is a bias-removing autoencoder, which allows the explicit trade-off between bias reduction and information loss. The second network is a feedforward network, which constructs effective treatment policies on the transformed data.

Task embedding-based causal effect variational autoencoder (TECE-VAE) scales CEVAE with task embedding to estimate the individual treatment effect using observational data for the applications that have multiple treatments (Saini et al., 2019). Additionally, TECE-VAE adopts the encoder-decoder architecture. The encoder network takes input **X** to generate distribution for **z**. The decoder network uses **z** to reconstruct features **X**, treatments *t*, and outcomes *y*. TECE-VAE uses information across treatments and is robust with unobserved treatments.

The conditional treatment-adversarial learning based matching method (CTAM) uses treatment-adversarial learning to effectively filter out the nearly instrumental variables for processing textual covariates (Yao et al., 2019b). CTAM learns the representations of all covariates, which contain text variables, with the treatment-adversarial learning, then performs nearest neighbor matching among the learned representations to estimate the treatment effects. The conditional treatment adversarial training procedure in CTAM filters out the information related to nearly instrumental variables in the representation space; therefore, the treatment discriminator, the representation learner, and the outcome predictor work together in an adversarial learning manner to predict the treatment effect estimation with text covariates. To be more specific, the treatment discriminator is trained to predict the treatment label, while the representation learner works with the outcome predictor to fool the treatment discriminator.

Reducing selection bias-net (RSB-net) uses two networks to address the selection bias problem (Zhang et al., 2019). The first net is an autoencoder that learns the representation. This autoencoder uses a Pearson correlation coefficient (PCC) based on regularized loss and explicitly differentiates the bias variables with the confounders that affect treatments and outcomes and the variables that affect outcomes alone. Therefore, the confounders and the variables affecting outcomes are fed into the second network, which uses the branching structure network to predict outcomes.

The variational sample reweighting (VSR) algorithm uses a variational autoencoder to remove the confounding bias in the applications with bundle treatments (Zou et al., 2020). VSR simultaneously learns the encoder and the decoder by maximizing the evidence lower bound.

### 4.4. Generative adversarial nets-based algorithms

Generative adversarial nets for the inference of individualized treatment effects (GANITE), as suggested by the name, infer the ITE based on the generative adversarial nets (GANs) framework (Yoon et al., 2018). The algorithm uses a counterfactual generate, G, to generate potential outcome vector ỹ based on features **X**, treatments *t*, and factual outcome *y*_*f*_. Then, the generated proxies are passed to an ITE generator that generates potential outcome ŷ based on feature **X**. As a generative adversarial net (Goodfellow et al., 2020), GANITE uses a discriminator for G, *D*_*G*_, and a discriminator for I, *D*_*I*_ to boost the training performance for the generators. *D*_*G*_ maps pairs (**X**, ȳ) to vectors in [0, 1]^*k*^ with the *i*−th component to represent the probability that the *i*−th component of ỹ is the factual outcome. Similarly, *D*_*I*_ maps a pair x, y^*^ to [0, 1] representing the probability of y^*^ being from the data $\tilde{D}$.

The causal effect generative adversarial network (CEGAN) utilizes an adversarially learned bidirectional model along with a denoising autoencoder to address the confounding bias caused by the existence of unmeasurable latent confounders (Lee et al., 2018). CEGAN has two networks: a prediction network (consisting of a generator, a prediction decoder, an inference net, and a discriminator), and a reconstruction network (a denoising autoencoder that has an encoder that is used as the generator in the prediction network).

SyncTwin constructs a synthetic twin that closely matches the target in representation to exploit the longitudinal observation of covariates and outcomes (Qian et al., 2021b). SyncTwin uses the sequence-to-sequence architecture with an attentive encoder and an LSTM decoder to learn the representation of temporal covariates and then constructs a synthetic twin to match the target in representations for controlling estimation bias. The reliability of the estimated treatment effect can be assessed by comparing the observed and synthetic pretreatment outcomes.

The generative adversarial de-confounding (GAD) algorithm estimates outcomes of continuous treatments by eliminating the associations between covariates and treatments (Kuang et al., 2021). First, GAD randomly shuffles the value of covariate **X** into **X**′ to ensure **X**′⫫*T*, where *T* is the treatments. Second, GAD reweights samples in **X** so the distribution of **X** is identical to **X**′. GAD then eliminates the confounding bias induced by the dependency between *T* and **X**.

Adversarial balancing-based representation learning for causal effect inference (ABCEI) uses adversarial learning to balance the distributions of covariates in the latent representation space to estimate the conditional average treatment effect (CATE) (Du et al., 2021). ABCET uses an encoder that is constrained by a mutual information estimator to minimize the information loss between representations and input covariates to preserve highly predictive information for causal effect inference. The generated representations are used for discriminator training, mutual information estimation, and prediction estimation.

### 4.5. Recurrent neural networks-based algorithms

The recurrent marginal structural network (RMSM) uses recurrent neural networks to forecast a subject's response to a series of planned treatments (Lim, 2018). RMSM uses the encoder-decoder architecture. The encoder learns representations for the subject's current state by using a standard LSTM to predict one-step-ahead outcome (Yt+2^) given observations of covariates and actual treatments. The decoder forecasts treatment responses on the basis of planned future actions by using another LSTM to propagate the encoder representation forwards in time.

The counterfactual recurrent network (CRN) uses a recurrent neural network-based encoder-decoder to estimate treatment effects over time (Bica et al., 2020b). The encoder uses domain adversarial training to build balancing representations of the patient's history to maximize the loss of the treatment classifier and minimize the loss of the outcome predictor. The decoder updates the outcome predictor to predict counterfactual outcomes of a sequence of future treatments.

The time series deconfounder uses a recurrent neural network architecture with multitask output to leverage the assignment of multiple treatments over time and enable the estimation of treatment effects in the presence of multi-cause hidden confounders (Bica et al., 2020a). The algorithm takes advantage of the patterns in the multiple treatment assignments over time to infer latent variables that can be used as substitutes for the hidden confounders. It first builds a factor model over time and infers latent variables that render the assigned treatments conditionally independent; then, it performs causal inference using these latent variables that act as substitutes for the multi-cause unobserved confounders.

### 4.6. Transformer-based algorithms

CETransformer uses transformer-based representation learning to address the problems of selection bias and unavailable counterfactual (Guo et al., 2021). CETransformer contains three modules, including a self-supervised transformer for representation learning, which learns the balanced representation, a discriminator network for adversarial learning to progressively shrink the difference between treated and control groups in the representation space, and outcome prediction, which uses the learned representations to estimate all potential outcome representations.

### 4.7. Multiple-branch networks and subspaces

The dose response network (DRNet) uses neural networks to estimate individual dose-response curves for any number of treatments with continuous dosage parameters (Schwab et al., 2020). DRNet consists of shared base layers, *k* intermediary treatment layers, and k*E heads for the multiple treatment setting, where *k* denotes the number of treatments and *E* defines the dosage resolution. The shared base layers are trained on all samples, the treatment layers are only trained on samples from their respective treatment category, and a head layer is only trained on samples that fall within its respective dosage stratum.

Disentangled representations for counterfactual regression (DR-CFR) disentangles the learning problem by explicitly identifying three categories of features: the ones that only determine treatments, the ones that only determine outcomes, and the confounders that impact both treatments and outcomes (Hassanpour and Greiner, 2019b). Three representation learning networks are trained to identify each of the three categories of factors, and the identified factors are fed into two regression networks to identify two types of treatments and two logistic networks to model the corresponding behavior policy.

Decomposed representations for counterfactual regression (DeR-CFR) disentangles the learning problem by explicitly dividing covariants into instrumental factors, confounding factors, and adjustment factors (Wu A. et al., 2020). DeR-CFR has three decomposed representation networks for learning the three categories of latent factors, has three decomposition and balancing regularizers for confounder identification and balancing of the three categories of latent factors, and has two regression networks for potential outcome prediction.

Neural counterfactual relation estimation (NCoRE) explicitly models cross-treatment interactions to learn counterfactual representations in the combination treatment setting (Parbhoo et al., 2021). NCoRE uses a novel branched conditional neural representation and consists of a variable number of shared base layers with k intermediary treatment layers, which are then merged to obtain a predicted outcome. The shared base layers are trained on all samples and serve to model cross-treatment interactions, and the treatment layers are only trained on samples from their respective treatment category and serve to model per-treatment interactions.

Single-cause perturbation (SCP) uses a two-step procedure to estimate the multi-cause treatment effect (Qian et al., 2021a). The first step augments the observational dataset with the estimated potential outcomes under single-cause interventions. The second step performs covariate adjustment on the augmented dataset to obtain the estimator. Curth and van der Schaar (2021) presented three end-to-end learning strategies for exploiting structural similarities of an individual's potential outcomes under different treatments to obtain better estimates of CATE in finite samples. The three strategies regularize the difference between potential outcome functions, reparametrize the estimators, and automatically learn which information to share between potential outcome functions.

Deep orthogonal networks for unconfounded treatments (DONUT) proposes a regularizer that accommodates unconfoundedness as an orthogonality constraint for estimating ATE (Hatt and Feuerriegel, 2021). The orthogonality constraint is defined as < *Y*(*t*)−*f*(**X**, *t*)>, *T*−*E*[*T* | **X** = *x*]), where $<\overset{•}{,}\overset{•}{>}$ is the inner product.

Subspace learning-based counterfactual inference (SCI) learns in a common subspace, a control subspace, and a treated subspace to improve the performance of estimating causal effect at the individual level (Yao et al., 2021b). SCI learns the control subspace to investigate the treatment-specific information for improving the control outcome inference, learns the treated subspace to retain the treated-specific information for improving the estimation of treated outcomes, and learns common subspace to share information between the control and treated subspaces to extract the cross-treatment information and reduce selection bias.

### 4.8. Combining DL with statistical regulators and kernels

The varying coefficient neural network (VCNet) puts forward a neural network and a targeted regularization to estimate the average dose-response curve for continuous treatment and to improve finite sample performance (Nie et al., 2021).

Shi et al. (2019) proposed the Dragonnet to exploit the sufficiency of the propensity score for estimation adjustment, and proposed the targeted regularization to induce a bias toward models. The Dragonnet uses a three-headed architecture to provide an end-to-end procedure for predicting propensity score and conditional outcome from covariates and treatment information. The targeted regularization introduces a new parameter and a new regularization term to achieve stable finite-sample behavior and strong asymptotic guarantees on estimation.

Deep kernel learning for individualized treatment effects (DKLITE2) is a deep kernel regression algorithm and posterior regularization framework to avoid learning domain-invariant representations of inputs (Zhang et al., 2020). DKLITE2 works in a feature space constructed by a kernel function to exploit the correlation between inputs and uses a neural network to encode the information content of input variables.

From the above introduction, it is clear that DL architectures are widely used in CL for reducing dimensionality, processing temporal data, balancing distributions, and removing confounding and selection bias. Among the architectures, autoencoder and GAN are particularly popular. From the application perspective, most of the above methods focus on estimating ITE, and the methods for estimating ATE and CATE do exist.

## 5. Deep causal learning for robotic intelligence

### 5.1. Challenges in intelligent robotics

Robotics is challenging. Robots are made up of many different components, including sensors, actuators, and control systems, all of which must work together seamlessly to function properly (Yoshikawa, 1990; Craig, 2005; Smith et al., 2020). Robotic systems are expensive to design, build, and maintain. Additionally, they are subject to regulations, standards, and certifications that need to be adhered to. These challenges require a multidisciplinary approach, combining expertise in areas such as mechanical engineering, electrical engineering, computer science, and cognitive psychology to design and build robots.

**Figure 3:**
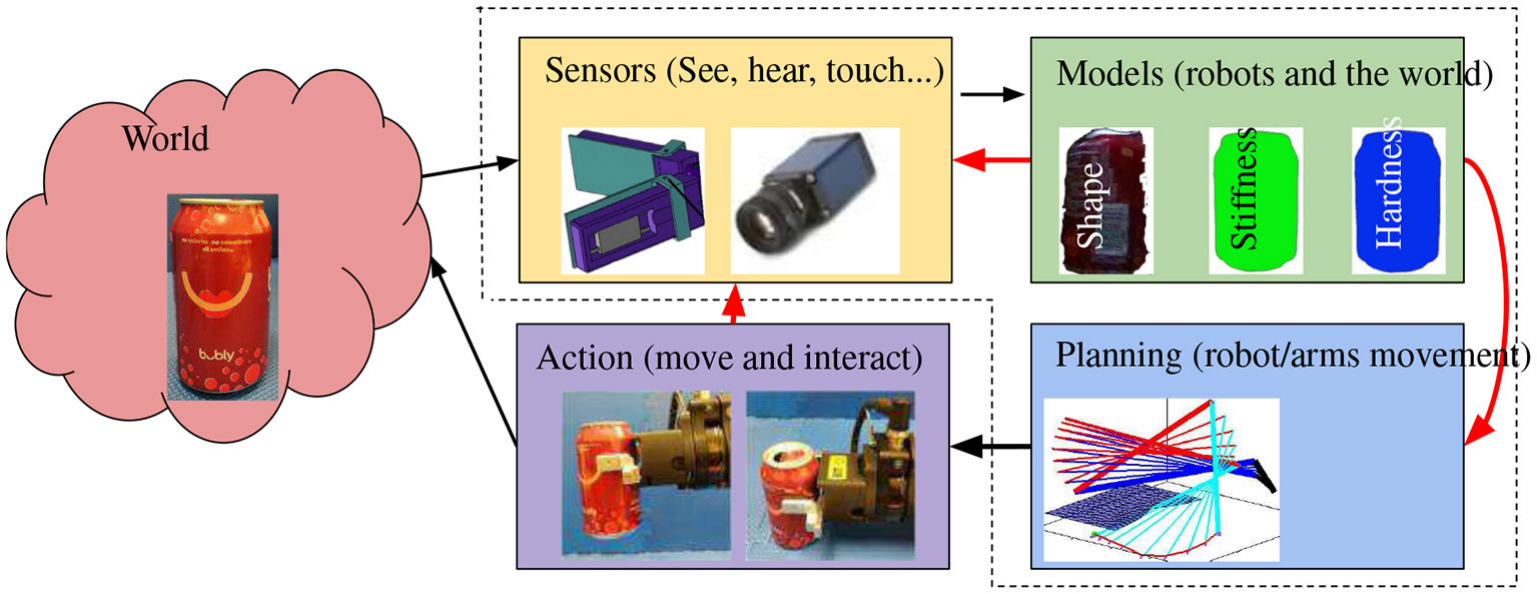
Intelligent robots observe to learn, plan for interaction, and revise to improve.

**Figure 4:**
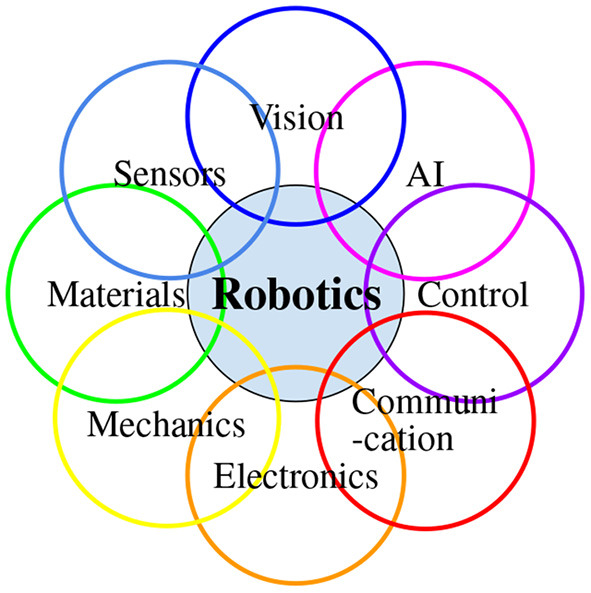
Robotics is multidisciplinary.

Intelligent robotics is built upon robotics and faces additional challenges as intelligent robots interact with the real world, which is subject to environmental uncertainties, sensory noises, modeling uncertainties, execution errors, and unexpected events (Figure 3). Intelligent robots are multidisciplinary by nature (Figure 4) and interact with humans in the real world, which is full of unexpected events. These facts introduce challenges that are being intensively studied.

- Perception: intelligent robots need to be able to perceive and understand their environment to navigate and interact with it. This includes tasks such as object recognition, localization, and mapping, which can be challenging due to the complexity of real-world environments and the presence of noise and uncertainty (Thrun et al., 2005; Li and Olson, 2010; Li et al., 2014, 2018e).
- Planning and decision making: robots need to be able to make decisions about how to move and interact with their environment, based on their perception of it. This requires the development of advanced algorithms for planning and decision making, which can be difficult to design and implement in real-world scenarios (Li et al., 2022; Liu et al., 2022a,b; Su et al., 2022b).
- Control and actuation: robots need to be able to execute the decisions made by their planning and decision-making systems by controlling their movements and interactions with the environment. This requires the development of robust control systems and actuators, which can be a challenging task, especially in highly dynamic and unpredictable environments (Li et al., 2012b, 2018d; Miyasaka et al., 2020; Qi et al., 2020; Su et al., 2022a).
- Interaction with humans: as robots increasingly interact with humans in shared spaces, it is important to ensure that they can understand human behavior and communicate effectively. This requires the development of human-robot interaction (HRI) algorithms and interfaces (Jin et al., 2018; Khan et al., 2018; Zhang et al., 2022).
- Understanding domain-specific knowledge: robots need to understand domain knowledge to accomplish tasks, such as robotic surgeries. This is especially challenging while the domain knowledge cannot be formalized as the rules and costs of collecting data are expensive (Li et al., 2017b, 2018a; Saxena et al., 2019). In such applications, robots need to learn from small data and adapt to various tasks (Li et al., 2018b, 2021).
- Safety and reliability: ensuring the safety and reliability of robots, especially in critical applications, is a major challenge. This requires the development of robust fault-tolerance and safety mechanisms, as well as rigorous testing and validation of the robot's performance (Li et al., 2013, 2017c; Alemzadeh et al., 2016; Li, 2019; Li Y. et al., 2019).
- Scalability: developing robots that can operate effectively in different environments and perform a wide range of tasks is a difficult challenge. This requires the development of modular and scalable robot systems that can adapt to different scenarios (Li et al., 2012a; Li S. et al., 2017; Majumdar et al., 2020).
- Ethical and societal concerns: as robots become more advanced and autonomous, there are ethical and societal concerns to be taken into account, such as potential job displacement, privacy, and security issues (Lin et al., 2014; Xu et al., 2022).

### 5.2. Deep causal learning for intelligent robots

While intelligent robotics has made significant progress in the past two decades, partially benefiting from the development of deep learning, intelligent robots are rarely used in real-world environments. Deep causal learning is a promising solution to the challenges of intelligent robotics in the real world. Deep causal learning infers the causal relationships and can provide a better understanding of the underlying mechanisms that generate the data, which can greatly improve safety and reliability, and make the ethical and societal concerns solvable. Deep causal learning models can be used to identify the most important factors that influence a particular outcome, thus simplifying the understanding of domain-specific knowledge. Deep causal learning can be used to generate counterfactual predictions, which can improve decision making and enable complimentary perception and understanding. Most importantly, deep causal learning extracts the structures of knowledge and enables stackable learning, which improves perception, control, and scalability.

To further explain how deep causal learning can potentially break bottlenecks in intelligent robotics, we use three examples, a low-level visual tracking example, middle-level motion planning, and high-level task planning to illustrate why we believe that deep causal learning has the potential to fundamentally change intelligent robotics.

**Figure 5:**
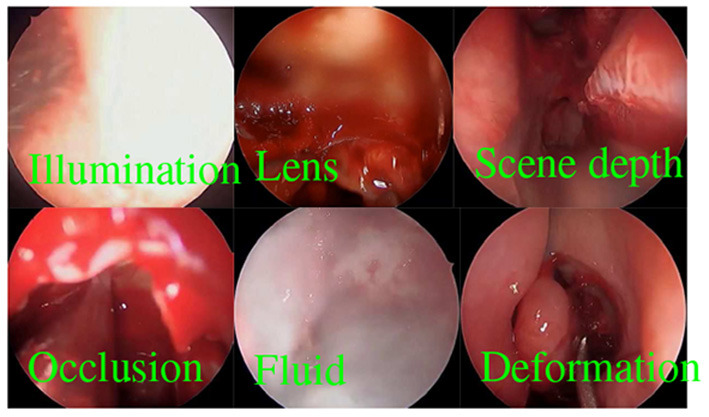
Visual tracking in endoscopic surgery.

Visual tracking is an important problem in robotics and is widely studied in the fields of computer vision, AI, and the robotics (Figure 5 and Table 1). There are a large number of results that can address the challenges of illumination changes, occlusions, lens blur, drastic scene changes, deformation, etc., particularly for visual tracking in endoscopic surgeries (Qin et al., 2019, 2020; Lin et al., 2020; Recasens et al., 2021). However, visual tracking remains challenging in endoscopic surgeries as all these adverse factors exist simultaneously and deteriorate tracking performance. Meanwhile, these adverse factors, along with the variance of pathology and anatomy, make the need for training data grow beyond our capacity. Therefore, we believe that deep causal learning is needed to disentangle the problems for robots.

**Table 1:** Visual tracking algorithms for addressing various challenges.

	**Sparse**	**Semidense**	**Full-dense**
Static	Davison et al. (2007), Mur-Artal et al. (2015)	Mur-Artal and Tardós (2015), Wu Y. et al. (2020)	Newcombe (2012)
Dynamic object	Saputra et al. (2018), Yu et al. (2018), Milford and Wyeth (2012), Pepperell et al. (2016)	Wen et al. (2020)	Wimbauer et al. (2021), Fehr et al. (2017), Bârsan et al. (2018)
Low texture	Yang et al. (2016), Gomez-Ojeda (2020)	Mahmoud et al. (2017)	Visentini-Scarzanella et al. (2017), Tateno et al. (2017), Ma et al. (2019), Lurie et al. (2017)
Image quality	Lee et al. (2011)	Mur-Artal and Tardós (2015)	Seok Lee and Mu Lee (2013), Chen et al. (2019)
Illumination	Whelan et al. (2016), Gomez-Ojeda (2020)		Mahmoud et al. (2016), Soper et al. (2012), Okatani and Deguchi (1997)
Failure recovery	Williams et al. (2007), Hsiao and Kaess (2019)		
Adverse motion	Vasconcelos et al. (2019)		Ma et al. (2019)
Deformation			Lamarca et al. (2020), Turan et al. (2017)
Scene depth			Péntek et al. (2018), Ma et al. (2019)

**Figure 6:**
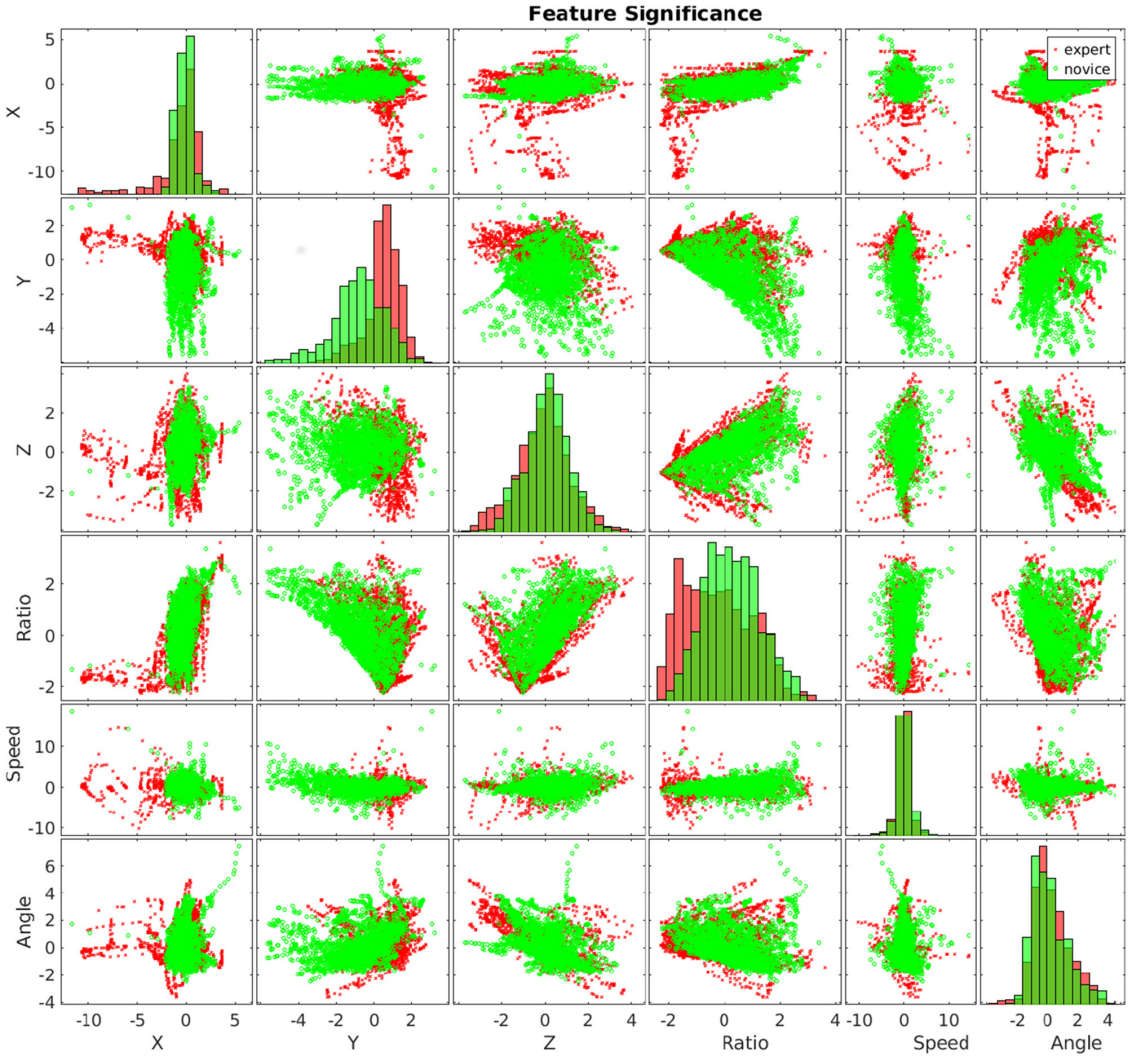
Experts (in green) and novices (in red) show significant differences in hand movements.

Motion planning is widely studied in robotics (LaValle and Kuffner, 2001; Li et al., 2018c). However, in real-world applications, intelligent robots not only need to know where to move to and how to move there but also need to know whether there are other application-specific requirements. For example, it has been well-studied that movement patterns impact surgical outcomes (Figure 6; Harbison et al., 2017; Li et al., 2017a), but it is not trivial to plan motions for a robot for various treatment procedures (Li and Hannaford, 2017, 2018). Therefore, we believe that deep causal learning, which naturally uses graphical structures to represent knowledge, can effectively incorporate domain knowledge with robotic techniques.

Task-level planning involves multiple decision-making and is specific to applications. For example, robotic surgery, as one of the most successful real-world applications of robotic technology, is still fully teleoperated, despite studies showing that many surgical accidents were caused by the incorrect operation of surgical robots (Alemzadeh et al., 2016; Su et al., 2020). Although we believe there are legal and regulatory barriers that prevent the adoption of autonomous technology, we argue that the main problem is that we lack the technology to handle environmental and task variance. For example, robots have problems dynamically adapting to changes and determining the completeness of surgery (Taylor et al., 2016).

## 6. Conclusion

Deep Causal Learning has recently demonstrated its capability for using prior knowledge to disentangle modeling problems and reduce data needs, improve performance at extrapolating unseen data, modularize learning problems, and incrementally learn from multiple studies. Inspired by these new findings, this Review incompletely but systematically discusses causal cognition, statistical causal learning, deep causal learning, and the need for deep causal learning in intelligent robots and argues that deep causal learning is the new frontier for intelligent robot research.

## Author contributions

YL organized the materials, wrote the manuscript, and contributed to the article and approved the submitted version.
